# Empathizing and Sympathizing With Robots: Implications for Moral Standing

**DOI:** 10.3389/frobt.2021.791527

**Published:** 2022-01-03

**Authors:** Oliver Santiago Quick

**Affiliations:** Research Unit for Robophilosophy and Integrative Social Robotics, Aarhus University, Aarhus, Denmark

**Keywords:** sympathy, empathy, HRI, moral status, phenomenology, social robot

## Abstract

This paper discusses the ethical nature of empathetic and sympathetic engagement with social robots, ultimately arguing that an entity which is engaged with through empathy or sympathy is engaged with as an “experiencing Other” and is as such due at least “minimal” moral consideration. Additionally, it is argued that extant HRI research often fails to recognize the complexity of empathy and sympathy, such that the two concepts are frequently treated as synonymous. The arguments for these claims occur in two steps. First, it is argued that there are at least three understandings of empathy, such that particular care is needed when researching “empathy” in human-robot interactions. The phenomenological approach to empathy—perhaps the least utilized of the three discussed understandings—is the approach with the most direct implications for moral standing. Furthermore, because “empathy” and “sympathy” are often conflated, a novel account of sympathy which makes clear the difference between the two concepts is presented, and the importance for these distinctions is argued for. In the second step, the phenomenological insights presented before regarding the nature of empathy are applied to the problem of robot moral standing to argue that empathetic and sympathetic engagement with an entity constitute an ethical engagement with it. The paper concludes by offering several potential research questions that result from the phenomenological analysis of empathy in human-robot interactions.

## 1 Introduction

Sympathetic and empathetic robots have become an increasingly popular topic of research within HRI. While a number of experiments have suggested that humans can feel sympathy or empathy for social robots (Riek et al., 2009; Leite et al., 2013; Rosenthal-von der Pütten et al., 2014; Leite, 2015; Ceh and Vanman, 2018; Menne and Schwab, 2018), the theoretical foundations of both the empathy and sympathy concepts, as well as their connections to ascriptions of moral standing, have been underexamined within the field of HRI. This paper will draw on philosophical, sociological, and psychological research to argue that not only are the concepts (and associated phenomena) of sympathy and empathy distinct, but that the tendency to employ one or both of these concepts without sufficiently clarifying in what sense they are intended has acted as a limiting factor on the progress of HRI research investigating these phenomena. To arrive at unified terminological standards is not only of importance for the comparability of HRI studies, however; as I shall argue here, it is also directly relevant for empirical and conceptual-normative research on the moral standing of robots.

I proceed in two steps. First, I will discuss three broad notions of empathy which researchers should have in mind when employing the concept, as well as offer a novel definition of sympathy that makes clear the distinction between empathy and sympathy and the connections of both phenomena to ascriptions of moral standing. Section two will briefly present the empathy and sympathy concepts, as well as discuss why the distinction matters and consider how the terms have been used within extant HRI research, while placing an emphasis on the valuable insights from phenomenological understandings of empathy—which have been insufficiently considered—and on the important empathy-sympathy distinction. The section three will turn to an analysis of the import sympathy and empathy can have on the moral standing of social robots. I will argue there that a phenomenological understanding of empathy suggests that empathetic or sympathetic engagement with a robot already constitutes an ethical engagement (i.e., engagement with the robot as one which possesses at least “minimal” moral standing). The approach to robot moral standing offered here is similar to, yet distinct from, the relational approaches to robot moral standing that have been offered by David Gunkel and Mark Coeckelbergh (Coeckelbergh, 2012; Gunkel, 2012; Gunkel, 2018a; Coeckelbergh, 2018), and is based primarily on the phenomenological understanding of empathy offered by Edith Stein (1964) and Max Scheler and Health (1923)).

## 2 Empathy and Sympathy

The term “empathy” has only existed in English for little over a century (Stueber, 2019), but the conceptual origins can be traced back at least to the 17^th^ century discussion of “sympathy,” where philosophers David Hume (Hume, 1740) and Adam Smith (Smith, 1759) leveraged the concept to explain a range of phenomena in human-human interactions that have since been further differentiated. While “empathy” has become increasingly popular as a broad label for “knowing what an Other feels” or “feeling what an Other feels,” the term “sympathy” has generally become understood as “feeling bad for an Other,” Even with these vague folk definitions of the two concepts, two issues with the usage of the terms in HRI research become immediately apparent. For instance, it becomes clear that there are at least two senses of “empathy,” and that sympathizing and empathizing are not the same thing. Additionally, and perhaps stemming from these conceptual issues, there is a lack of sufficient conceptual care in relating empathy and sympathy to ascriptions of moral status, as will be elaborated in what follows. In this section, I will begin by discussing the two senses of empathy already suggested, as well as a third, more “basic” sense, before presenting a sympathy definition that captures the distinction between empathy (in all three senses) and sympathy.

### 2.1 Empathy: Cognitive, Affective, and Phenomenological Understandings

The first form of empathy, “knowing what an Other feels,” is often discussed under the label of “mind-reading” (Goldman, 2006; Singer, 2006), “mentalizing” (Singer, 2006) or “cognitive empathy” (Stephan, 2015; Bloom, 2018). Cognitive empathy can be understood as a process by which we are attribute mental or affective states to an Other, but do not “share” in these states or feel them ourselves. For instance, in seeing a stranger crying, I might infer that they are sad—if so, I have cognitively empathized. I need not feel sad myself in order to reach this conclusion, nor need I care about the sadness of the stranger. Such inference-based empathizing can be understood, broadly, as falling under the “theory of mind theory” (Carruthers and Smith, 1996) understanding of how empathy occurs. On the other hand, I might also simulate, or use my imagination, to attribute the sadness in one of two possible ways. Firstly, I might imagine what would make me cry in a public setting and decide that what the crying stranger is experiencing it is most likely sadness. Secondly, if the person is someone I know well rather than a stranger, I might imagine what would make *him* cry in a public setting (i.e., by taking into account information about his attitudes, beliefs, etc.,). In either case, as in inference-based cognitive empathy, I will not feel sad myself, nor need I care about the Other for the empathy to succeed.

Indeed, it is constitutive of cognitive empathy that I do *not* feel what the Other feels, for in the case where I “share” the affect of the Other (sadness, in this case), I am actually *affectively* empathizing. Like cognitive empathy, affective empathy is typically understood as relying on either inferences or simulations, but with the addition that one experiences an affective state similar to that of the Other. For instance, my understanding and “sharing” of a rock-climber’s fear can arise through my connecting aspects of her situation to affective memories of my own (Adams, 2001). Alternatively, this can also occur through imagining myself in the climber’s situation (Ravenscroft, 1998), or supposing that the climber is not a stranger, through a simulation of what I believe she is likely to be experiencing. While affective and cognitive empathy are clearly distinguishable by the inclusion or exclusion of “state-matching,” current HRI research tends to employ the term “empathy” without defining the term or in such a way that the boundaries between affective empathy, cognitive empathy, and sympathy become blurred.

For instance, consider a 2018 study by Ceh and Vanman, where “empathy” was measured with the two response items “I think this scenario is sad” and “I would have sympathy for someone in this situation” (Ceh and Vanman, 2018, p. 11). Believing a scenario to be sad is not the same as empathizing with a particular social agent. Likewise, sympathizing with someone goes above and beyond empathizing with them, as will be argued in the following section. Similarly, a 2009 study by Riek et al. investigating “empathy” for robots with distinct degrees of human-likeness measured the “empathy” of participants for the robots in terms of sympathy: “After each of the clips, we asked respondents a single question, ‘How sorry do you feel for the protagonist?’” (Riek et al., 2009, p. 4). When they compared the results of this question to their baseline measurements of dispositional empathy—which was measured via the Empathy Quotient (EQ) (Baron-Cohen and Wheelwright, 2004)—the researchers found that higher scores on the EQ did *not* predict higher “empathy” as measured by their single question. The lack of confirmation of their hypothesis is not surprising given the way they chose to measure empathy; the EQ is largely directed at perspective taking (e.g., “I am good at predicting how someone will feel” (Baron-Cohen and Wheelwright, 2004, p. 172)) and social intelligence (e.g., “I can easily tell if someone wants to enter a conversation” (ibid. 171)). However, despite the majority of the questions on the EQ targeting “empathy,” the developers of the metric actually intentionally included aspects of sympathy (e.g., pity, compassion, and concern), simply because they see sympathy “as a clear instance of the affective component of empathy,” which includes a motivation to help (Baron-Cohen and Wheelwright, 2004, p. 164).

This indicates that the tendency to treat empathy and sympathy as interchangeable is not limited to HRI but represents a much larger trend, which has simply been carried over. Indeed, there are some accounts of “empathy” which employ the term in a very broad sense, encompassing affective and cognitive empathy, sympathy, compassion, emotional contagion, and a variety of other interpersonal phenomena (e.g., Preston and de Waal, 2002). The desire to adopt a definition of empathy that encompasses all of these related interpersonal phenomena is understandable, of course. Indeed, as Frederique de Vignemont and Tania Singer suggested, “There are probably nearly as many definitions of empathy as people working on the topic” (de Vignemont and Singer, 2006, p. 435). Unfortunately, such approaches to defining empathy directly conflict with what was meant by “empathy” when the term was first coined, as well as what is frequently meant by terms such as “compassion” and “sympathy.” The distinction between cognitive empathy (“mentalizing”) and affective empathy, for instance, is not merely a matter of terminology, but also of physiology (Singer, 2006). From a phenomenological perspective, it is also clear that a phenomenon such as emotional contagion, for instance, is explicitly *not* “empathy” (Scheler and Health., 1923; Stein, 1964; Zahavi, 2014).

The affective/cognitive empathy distinction, and the distinction between sympathy and empathy, are perhaps still underutilized, but have begun to receive attention within HRI (Asada, 2015; Stephan, 2015; Quick, 2020). However, a third *phenomenological* understanding of empathy has been largely overlooked within HRI. As I will argue, this sense of empathy, which can be more or less understood in terms of what Alvin Goldman has called “low-level mindreading” (Goldman, 2006) or Karsten Stueber calls “basic empathy” (Stueber, 2006) is perhaps the most important for the question of moral standing. Basic empathy, as opposed to “complex empathy” (Hollan, 2012) (i.e., affective and cognitive empathy processes), is an automatic process wherein the Other is given as experiencing, and often as experiencing a particular state. That is, rather than taking an object of perception and imagining or inferring my way to what it might be experiencing, I actually “directly perceive” (Zahavi, 2014; Zahavi and Rochat, 2015) its experience. For example, upon seeing a man crying, I might simply “perceive” that he is sad, without engaging in more conscious (“complex”) empathy processes.

From a phenomenological perspective, empathy is fundamentally “how we experience others” (Zahavi, 2014, p. 130); it is the act “in which foreign experience is comprehended” (Stein, 1964, p. 6). Furthermore, this ‘basic’ class of empathy is a necessary precursor to, or component of, simulation-based and theorization-based complex empathy processes. In both instances—whether I am imagining or inferring the state of a target entity—I must first “grasp” the entity as an Other that is capable of experience. Indeed, I cannot be said to empathize with an entity unless I have already engaged with it as an experiencing Other, for—as the phenomenological perspective illustrates—empathy is precisely the experiencing of foreign experience. This “basic” or phenomenological class of empathy not only underpins the more “complex” forms, but as will be argued in section 2.2, it is also a necessary component of sympathy. In section 3 of this paper I will argue that the basic kind of empathic engagement described here is an ethical engagement, such that in empathizing with an entity,^1^ we have already engaged with it as possessing “minimal” moral status. Of the three forms of empathy discussed here, the phenomenological account has received the least attention in HRI.^2^ However, HRI research on robot emotion expression (Kühnlenz et al., 2013; McColl and Nejat, 2014) could be understood as falling under the umbrella of basic empathy, in that the researchers aim to prompt users to perceive robots as experiencing certain states.

### 2.2 Defining Sympathy

As indicated in the section 2.1, sympathy is generally understood as feeling bad “for” an Other. Because of this, it is often conflated with pity—a term that has in recent history acquired a negative connotation (Nussbaum, 1996). “Pity” and “compassion,” though not directly discussed in this paper, are closely related to sympathy—pity is best understood as a reduced form of sympathy, while “compassion” can be understood as describing a particularly strong instance of sympathy (Quick, 2021). However, upon closer examination, sympathy is a complex phenomenon that is closely related to compassion (Nussbaum, 2001) and is subject to complex social and interactional norms (Clark, 1997). Thus, I offer the following definition of sympathy:^3^ (Quick, 2021):

Sympathy is a prosocial response **R** to the negative situation of an Other, which leads to an altruistic motivation, and whose appropriate expressions and instantiations are context-dependent and governed by dynamic social norms. **R** consists of several components, the first five of which are necessary, while the sixth is facultative:

 i) Sentiment ‘for’
 ii) Some level of empathizing
iii) A judgment of seriousness
 iv) A non-fault judgment
 v) A value judgment

In addition, **R** may include:

vi) A specific behavioral display

In what follows, I will briefly^4^ argue for the necessity of each of the components, beginning with the claim that sympathy is “subject to complex social and interactional norms.” Candace Clark’s extensive sociological research of American sympathy norms (Clark, 1987; Clark, 1997) suggests that sympathy is best understood in terms of *exchanges*—giving sympathy places an obligation of repayment on the Other, just as accepting sympathy places an obligation of repayment on oneself. Furthermore, sympathy exchanges need not occur in a one-to-one, universalizable fashion, they are instead always situated within a specific social context that dictates acceptable forms of displaying and repaying sympathy, as well as which sorts of circumstances merit sympathy. Sympathy “costs the donor time, effort, and emotional energy” (Clark, 1997, p. 130), and is thus a valuable commodity in our socio-emotional economy. Displays of sympathy are, as Arie Hochschild’s work on emotions suggests, a form of “emotional labor” that is governed by “display rules” (Hochschild, 1983, p. 60). To be an effective sympathizer, one must understand—and comply with—the local sympathy norms.^5^ A social agent that fails to act in accordance with these norms may be seen as what Clark has called a “sympathy deviant” (Clark, 1997, p. 22), eventually resulting in exclusion from the sympathy network.

The similarities between sympathy and compassion can be found in components (iii-v), which are drawn (and modified) from Martha Nussbaum’s Aristotelian account of compassion, as well as Daniel Batson’s social-psychological account of compassion (Batson, 2011). The judgment of seriousness indicates that for *genuine* sympathy to occur, the sympathizer must judge that the suffering of her target must be non-trivial or significant in some fashion. For instance, the suffering incurred by a paper cut is typically not seen as worthy of sympathy as the suffering incurred by losing a loved one. The non-fault judgment indicates that a sympathizer must judge that the victim is not responsible for his plight, or that if he is responsible, some extenuating circumstances mitigate this responsibility. Suppose I break my hand punching in a car window—without further information, my plight merits only minimal sympathy, if any. However, if we add that I punched in the window to rescue a baby who had been left in the hot car with closed windows for several hours, despite my being responsible for my injury, the altruistic intention behind the act can mitigate the importance of the fault, such that an observer may be more inclined to sympathize with me.

Finally, the value judgment (“eudaimonistic” judgment, in Nussbaum’s terms (Nussbaum, 2001)), indicates that the object of one’s sympathy must be seen as relevant to one’s own flourishing—it must be “a significant element of my scheme of goals and projects, an end whose good is to be promoted” (Nussbaum, 2001, p. 321). Alternatively, in Batson’s phrasing, I must “care about whether the other is in need and about how this need affects the other’s life” (Batson, 2011, p. 41). Thus, in genuinely sympathizing, I will have judged (perhaps implicitly) that the suffering of the Other is serious, not of his or her own making (or justifiably so), and that the Other matters to me in some fashion. This “mattering” can take various forms. For instance, I need not explicitly judge that the Other—say, a robot who is being mistreated—is actually suffering, or indeed actually capable of suffering, but only that the robot appears to be suffering, while holding as a part of my ‘scheme of goals and projects’ a belief along the lines that “suffering is bad.” As such, any entity which is perceived as suffering could be seen as relevant for my flourishing and judged as having value (at least initially—that is to say, judgments are subject to revision). It is here that perceptual, or phenomenological, empathy plays a particularly important role, in that it accounts for how we can perceive an entity (a robot, human, animal, etc.,) as suffering. For this reason, I argue that empathy “in some form” 2) is also a necessary component of sympathy—one cannot genuinely sympathize without first perceiving (or judging via inference or simulation) that the entity in question is suffering in some sense.^6^ Likewise, it is constitutive of sympathy that one “feels for” 1) the victim. If I do not on some level feel (e.g., bad, or sad) for the victim, I cannot be said to genuinely sympathize.^7^

The sixth, facultative component of sympathy (display) is likely the most important in terms of human-robot sympathy exchanges, in that it seems to be, currently, the easiest and most impactful of these components to equip social robots with. Sympathy displays can take both what might be called *overt* and *subtle* forms. Overt displays of sympathy include acts such as verbal affirmations of sympathy (“I am sorry to hear that”), the giving of gifts such as flowers or money, or acts such as attending a funeral with a friend who has lost a loved one. Subtle displays, on the other hand, encompass acts such as sympathetic facial expressions or physical contact (e.g., placing a hand on a victim’s shoulder). However, because of the complex and socially relative norms that govern when and how to express sympathy, sympathy displays can be seen as constituting the adoption of a social, political, or moral stance (i.e., by showing that one believes this particular plight is indeed worthy of sympathy). For instance, expressing sympathy for a woman who is unable to receive an abortion in Texas due to the 2021 anti-abortion legislation can be interpreted as adopting a “pro-choice” stance. Thus, the situations which merit sympathy displays, and the manners in which social robots ought to display sympathy—particularly in cross-cultural contexts—will need to be carefully considered (Quick, 2020).

Already in light of the preceding brief discussion of empathy and sympathy, it has become clear, I hope, why it is urgent that the concepts are employed with further care than is often seen in HRI. An experiment that measures empathy in cognitive terms is not immediately comparable to an experiment which measures empathy in affective terms, nor are they comparable to an experiment that purports to measure empathy but actually measures sympathy. In addition, more careful attention to the cognitive and emotional processes involved in these two different phenomena, empathy and sympathy, can prove decisive for the discussion of whether robots can or should have moral standing, and ensuing recommendations for the (physical-kinematic and functional) design of the robot. While the problem of mixing empathy and sympathy is not unique to HRI research, the increasing interest in commercial and domestic social robots lends an urgency to the task of understanding the social and moral implications of social robots that elicit or display sympathy and empathy that is simply not found in many areas of research. For instance, whether philosophers and psychologists agree on the nature of empathy and sympathy in 10 years or in one hundred years makes relatively little difference in practical terms. Such research may indeed result in social benefits (e.g., improved techniques in therapy or pedagogy), but a failure to reach conclusions here, or a delay in doing so, will at least not actively cause harm. The same cannot be assumed in the case of sympathetic and empathetic robots. Because the development of such devices is still in early stages, it is not clear what ethical, social, or emotional impact such devices may have on their users.

## 3 Robot Moral Standing

I will argue in this section that empathizing—in any of the three senses discussed—involves an ascription of what we might consider “minimal” moral standing, while sympathizing involves a still greater ascription of moral standing. The argument for why empathizing with a robot entails an ascription of moral status can be seen as proceeding from four premises (Quick, 2021), each of which I will argue for by drawing primarily on the works of the phenomenologists Edith Stein (1964) and Max Scheler (1923). These are:

1. The feelings a human may have for, or on behalf of, a robot are genuine experiences of the same kind as those a human may have for, or on behalf of, another human, regardless of the robot’s (lack of) internal states.
2. The human experience of foreign experience or, more precisely, of “an experiencing Other,” is^8^ of one kind, regardless of the ontological status of the Other.
3. Actions perceived as intentional are apprehended as originating from an experiencing Other.
4. Others apprehended as experiencing are due moral consideration.

### 3.1 Experiencing Otherness and Moral Standing

The first premise can be traced to Edith Stein’s account of empathy, wherein she argued that while we may be deceived with regards to the object of our feelings, we cannot be deceived as to the existence of the feelings themselves. “I can be deceived in the object of my love, i.e., the person I thought I comprehended in this act may in fact be different, so that I comprehended a phantom. But the love was still genuine” (Stein, 1964, p. 31). In other words, even though the object of my love was not reciprocating the feeling, was unfeeling with respect to love or not as I initially comprehended it to be, my own feeling of love towards the Other was still genuine. With this, we can understand the results of HRI research such as Bartneck and Hu’s 2008 Milgram experiments, wherein the researchers noted that “the participants showed compassion for the robot” (Bartneck and Hu, 2008, p. 420).^9^ The sympathy (or compassion) that participants felt towards the robotic victim was of the same kind that participants in the original Milgram experiment might have felt for the human victims, and just as genuine, regardless of the fact that the robots were not actually suffering. Thus, even if a participant came to know that the robot was not actually suffering (and was in fact incapable of suffering), the empathic experience he or she had of the Other as “experiencing pain” remains genuine.

This leads directly to the second premise, which argues that our experience of an entity as an Other that is experiencing (or is capable of experiencing) mental or affective states is not tied to the actual or perceived ontological status of that entity. In other words, whether a robot is actually capable of suffering or not—or whether the observer believes it to be capable of suffering or not—it is entirely possible for one to experience the robot as suffering. The insights of early phenomenologists that “experiencing X as suffering” is independent of the ontological state of X also seems to underlie Mark Coeckelbergh’s argument that “whatever the ‘real’ status of the robot may be, it is its appearance that is relevant to how the human-robot relation is experienced and constructed” (Coeckelbergh, 2011, p. 198). Unsurprisingly, given his use of the phenomenology of Emmanuel Levinas, a similar thread can be found in David Gunkel’s work, when he argues that rather than first identifying the ontological status of an entity and then deciding on its moral status, “we are first confronted with a mess of anonymous others who intrude on us and to whom we are obligated to respond even before we know anything at all about them” (Gunkel, 2018b, p. 96). In sum, the first and second premises can be taken as suggesting that the human empathic experience of otherness (i.e., the experience that a particular Other is capable of experience or is experiencing an affective or mental state) is not contingent on ontological knowledge and is, as such, not to be understood as a perceptual mistake—as experience, it is a correct processing of the data (Quick, 2021, p. 258).

Of course, some objects and entities might lend them themselves to being experienced empathically as Other more readily than others. Two possible reasons for this are as follows. First, it could be that there are certain affordances or characteristics that we recognize in objects as being associated with Otherness. For instance, Stein discusses what she calls “the specific phenomena of life,” which include “growth, development and aging, health and sickness, vigor and sluggishness” (Stein, 1964, p. 68). As she indicates, it is not merely that we attach these characteristics to an object after perceiving it, but rather, that through the act of empathy they are “co-seen”— “Thus, by his walk, posture, and his every movement, we also “see” “how he feels,” his vigor, sluggishness, etc.” (Stein, 1964, p. 69). Certain objects, such as humans, animals, and social robots, simply present themselves as experiencing these states more clearly than objects such as rocks or guitars. Additionally, another key difference between objects such as rocks and robots is simply the fact that social robots (often) possess some movement capabilities. More precisely, they can present themselves as capable of voluntary movement in a way that rocks simply cannot.^10^ In line with this, a second reason for why we empathize more readily with some objects than others could have to do with similarity to previous Others. That is, if an object is similar to, shares sufficient characteristics with, or in some meaningful way reminds me of one that I have previously grasped as Other, I may be predisposed to grasp it as such than if it did not. For example, a humanoid robot may be more readily grasped as Other simply because it bears a resemblance to the “standard” Other—humans. A rock, on the other hand, does not bear much of a resemblance to humans, or animals, or social robots—thus, it may be less predisposed to grasping it as Other through empathy.

The third premise holds that actions which are perceived as intentional are perceived (perhaps implicitly) as originating from an experiencing Other. That is, if we understand an action as intentional, then we are understanding it as an action that is underpinned by a volition, intention, or willing.^11^ While the nature of these three concepts is debatable, they are all undoubtably experiential in some sense, such that an agent which is incapable of experiencing is incapable of willing or having intentions or volitions in the way that humans are. Despite believing this, we often engage with agents—such as social robots—*as if* they are acting intentionally,^12^ or *as if* they are experiencing. Regardless of whether (or not) participants explicitly believe a social robot possesses mental states, intentions, or experiences, humans often seem to engage with them as if they do, going so far as to feel bad for them when they are “suffering” (Bartneck and Hu, 2008; Darling et al., 2015; Seo et al., 2015; Carlson et al., 2019; ). If it is the case that intending and willing are a form of experiencing, then we can see that robotic actions which are perceived as intentional are perceived as originating from an experiencing Other—for, as Scheler wrote, “…we cannot be aware of an experience without being aware of a self…” (Scheler and Health, 1923, p. 9). With respect to the current discussion, we could modify this to say that “we cannot perceive an experience without perceiving an Other” Similarly, Stein argued that “willing is essentially motivated by a feeling” (Stein., 1964, p. 97) and “the foreign person is constituted in empathically experienced acts. I experience his every action as proceeding from a will and this, in turn, from a feeling” (ibid. 109).

The fourth premise is a normative claim that any entity which is apprehended as an experiencing Other is due some level of moral consideration. It is with regards to this claim in particular that the phenomenological account of empathy offers something novel to the current debate about robot moral standing, which has largely centered around the Kantian, utilitarian, and virtue ethics based answers to the problem.^13^ The phenomenological perspective offers an epistemological argument—in not opening ourselves to the full datum (experiencing the Other as experiencing *and* worthy of moral consideration) we are making an experiential mistake. That is, a robot simulating experiential states is “correctly” experienced when it is experienced as an experiencing Other, and qua this, also as due moral consideration. The argument can be framed in terms of Scheler’s discussion of brutality, which is understood as the “disregard of other peoples’ experience, despite the apprehension of it in feeling” (Scheler and Health, 1923, p. 14). Furthermore, “to regard a human being as a mere log of wood and to treat the object accordingly is not to be “brutal” towards him” (ibid.)—we are only brutal in cases where we apprehend an entity as an experiencing Other yet do not extend moral consideration to it. If an object is genuinely seen as an unintentional, non-experiencing object and treated as such, then it seems we are not engaging in brutality. Likewise, when a robot is genuinely experienced as non-experiencing, as non-Other, not including it in our moral considerations is not a moral failure, it is a correct processing of the data. The situation under consideration here is one where the robot or entity *is* experienced, through empathy, as an Other—it is here that moral consideration is due.^14^ However, this brings us to a second, closely related and similarly morally objectionable act in which we deny the experience of an entity; namely, dehumanization. In dehumanizing a person, we may ascribe fewer human attributes to them, or go so far as to ascribe “deficient or absent humanity to a target” (Haslam and Loughnan, 2014, p. 406). In regarding a human as a “mere log of wood,” we are dehumanizing her, stripping away her experiencing otherness—we are not brutalizing her, for we did not first empathically experience her as an experiencing Other.^15^

Dehumanizing is clearly morally objectionable for various reasons, but I will argue that one of the factors that makes it “wrong” is related to that which makes brutality wrong—the denial (or disregard) of experiencing otherness. To further investigate this claim, we can adopt a distinction between two types of capacities: “agency” (i.e., cognitive capacities such as planning and thought) and ‘experience’ (i.e., capacities such as emotions and consciousness) (Gray et al., 2007). While Gray et al. used “experience” to indicate a specific set of capacities which are distinct from those that fall under the “agency” category, per the arguments discussed in relation to the third premise, it appears that agentic capacities are experiential. For instance, take the standard understanding of “thinking”—this can be understood as being an intentional act, or as motivated by the will or feelings directly, or as a phenomenal act, in that there is there is “something that it is like” to “think.” When we say a non-human object, such as a robot, is “thinking,” we are either simply using figurative language, or anthropomorphizing the robot. In the latter case, we are perceiving the robot as an experiencing Other and, according to the preceding arguments, we have incurred an obligation to extend at least some moral consideration to the robot. Such a position is compatible with, and provides further support for, virtue-based arguments for extending moral consideration to robots. On such a view, “mistreating a robot is not wrong because of the robot, but because doing so repeatedly and habitually shapes ones moral character in the wrong kind of way… Mistreating the robot is a vice” (Coeckelbergh, 2018, p. 145).

As suggested earlier, the experience of foreign experience (i.e., empathy, in the phenomenological sense) is not merely a perceptual mistake; rather, it is a way of being “true to the situation.” Incorporating a phenomenological perspective of empathy thus introduces a methodological switch for the discussion of robot moral standing. Instead of considering the acts of the subject in relation to a preconceived ontology of the object, and thereby sorting our perceptions as “accurate” or “inaccurate,” the phenomenologist analyzes “what is given in experience.” On such a view, we can see that what occurs in brutality and dehumanization is a failure to take in fully that which is “there for experiencing.” Social robots that simulate experiential states can create the same sort of experiential data as humans do, and a rejection of this data is an experiential (and ethical) error of the same sort that brutalizing or dehumanizing a human would be—it is a rejection of the “phenomenological truth” which confronts me.

### 3.2 A Return to Sympathy

From the discussion of sympathy and empathy found in the previous sections, it seems that sympathy is of greater importance for the debate of robot moral standing than empathy is (particularly in terms of cognitive and affective empathy). For one thing, sympathy includes empathy as a necessary component, such that if I sympathize with a robot, I have already empathized with it. At this point, I have already framed the robot as an experiencing Other and ascribed a “minimal” moral status, in that I have incurred an obligation to take the experiential data given through my empathy seriously. Sympathizing with a robot, however, requires that I engage with it as an experiencing Other to an even greater extent. I must consider whether the robot’s “suffering” is of its own making, whether it is serious, and perhaps most importantly for questions of moral standing, I must judge the robot (or it’s suffering) as important and relevant to my own flourishing in some sense. It is unsurprising then that the focus of current HRI research on sympathy in human-robot interactions has typically been on whether humans can have sympathy for robots. While this is certainly an important question, the discussion does not move the robot beyond the status of a potential moral patient. An investigation of situations in which a robot shows sympathy for a human, on the other hand, would move us into the realm of considering whether robots can be potential moral agents.

A robot which displays sympathy for a human is potentially a moral agent in that it presents itself as an entity that is capable of experiencing foreign experience, as well as one which understands the local sympathy norms (at least in so far as it is able to comply with them). Despite the importance of investigating sympathy displaying robots, the topic has received little attention,^16^ perhaps in part due simply to technological limitations. A robot which is able to display sympathy convincingly or meaningfully will require reliable affect recognition as well as a set of rules for the sorts of situations that require displays and a library of potential displays that are linked to specific classes of situations. Indeed, as Kerstin Fischer suggests, “When we speak of robots processing and using social signals, then we are discussing future technologies” (Fischer, 2019, p. 19). The investigation of what sort of status is due to a robot which displays sympathy raises a variety of questions for future research. For instance, Clark’s research suggests that sympathy requires reciprocity, such that we can predict that when we sympathize with a robot (in the context of a long-term interaction), we will eventually expect ‘repayment’. In human-human relations, one of the principal forms of repaying the sympathy someone offers you is with an offer of sympathy (at a future, appropriate, time). Thus, an effective sympathetic robot, for instance, one that is intended to act as a “companion,” will require the ability to offer sympathy as well as accept it if it is to function as an effective actor within our sympathy networks (Quick, 2020). Indeed, if Clark’s observations regarding the expectation of reciprocity in sympathetic interactions between humans holds true in the case of robots, we should investigate what sort of threat a robot which elicits sympathy—without sympathizing in turn—poses to our sympathy conventions.

As argued in the previous section, when we sympathize with a robot, we are not making a “sentimental mistake” rather, we are avoiding precisely such an experiential mistake (brutalization or dehumanization) by being open to the available phenomenological truth. However, when it comes to a robot’s display of sympathy, we must ask whether this “truth” is present in the same manner—are we experiencing a foreign experience of foreign experience in the way that we can with a human’s display of sympathy? That is, can a robot’s apparent sympathy for a human be empathically experienced as genuine—in the way that data from HRI has suggested that a robot’s suffering can—or will it always be perceived as a simulation of sympathy? In sum, the ethical debate about the moral standing of robots appears to be miscalibrated. The focus should not be on whether Kantian or virtue-ethical arguments are better for justifying “attributions” of moral standing, but should rather be on: how much do we want to threaten our sympathy conventions? Our empathic engagement with the robot already indicates an ethical engagement with it, in that we have experienced it as an experiencing Other. Is it preferable to have social robots that we can genuinely sympathize with—to open ourselves to what is given in experience, the datum of foreign experience—but which will not show sympathy? Or should robots which elicit sympathy also show sympathy, even though it may be perceived as inauthentic? These questions are very different than those which are typically discussed in relation to robot moral standing and are of a more empirical than normative nature.

## 4 Conclusion

In this paper, I have argued that the debate over empathy in human-robot interactions has largely failed to recognize the distinctions between the three types of empathy on the one hand, and sympathy on the other. Furthermore, the phenomenological account of empathy, which offers critical insights and valuable research avenues into the question of robot moral standing, has largely been overlooked. This type of empathy, namely empathy as the “experience of foreign experience” is not only central to other forms of empathy (such as affective and cognitive empathy) as well as sympathy, but also explains best the connection between empathy, sympathy, and moral standing. Additionally, I argued for a novel account of sympathy which attempts to clarify the distinction between empathy and sympathy and outline the necessary conditions for an instance of genuine sympathy. In relation to this, two types of experiential errors—brutality and dehumanization—were discussed, and it was argued that both represent a failure to properly consider the data provided through our empathetic and sympathetic experiences. While the phenomenological analysis of empathy and the account of sympathy that have been discussed here offer a way of reframing the question of robot moral status, they also lead to a wide range of new questions for HRI research, several of which were posed in section three. Recognizing that our empathic engagement with an Other already also constitutes an ethical engagement with it allows for us to move from the heavily discussed normative questions to novel ones, as well as conduct empirical research on to what extent humans feel and respect the moral obligations which result from engaging with social robots that display (and elicit) differing levels of affect and sympathy.

## Footnotes

1 Note that because both complex empathy processes and sympathy are built upon this basic empathy process, the ethical engagement carries over into such interactions.

2 This is not to say that phenomenological accounts of empathy have received *no* attention in HRI, for instance (Coeckelbergh, 2018) has also engaged with empathy, phenomenology, and robot moral status. As indicated in the introduction, I believe the approaches are compatible. Indeed, the conclusions reached by Coeckelbergh (and Gunkel, for that matter) are highly similar to those offered here, although the means of reaching these conclusions is different. The three approaches all emphasize that the phenomenology of human-robot interactions should be taken seriously. This account contributes to the discussion primarily in terms of an analysis of empathy and sympathy that supports the importance of the phenomenology of human-robot interactions and a reframing of the discussion in terms of implications for the design of empathetic and sympathetic robots.

3 This definition of sympathy is drawn from (Quick, 2021).

4 The argumentation for this account of sympathy is per force brief, as the focus of this paper is on the implications sympathy and empathy have for robot moral status. For an extended discussion of various notions of sympathy see (Quick, 2021).

5 For a further discussion of the norms and how they affect the design of sympathetic social robots, see (Quick, 2020).

6 While Nussbaum has argued that empathy is not necessary for compassion, this seems to be because she limits the type of empathy considered to one that functions via simulation or imagination (Nussbaum, 2011, p. 149).

7 One can also feel what might be called “routinized” sympathy, where one might have felt sympathy for an entity in the past but due to repeated expose no longer holds the judgments or sentiment in an “active” sense. For instance, I might actively sympathize with a homeless man the first time I see him on my way to my office, but over time come only to feel this “routinized” sympathy—active sympathy requires, as Clark indicates, “time, effort, and emotional energy” (Clark, 1997, p. 130).

8 The usage of “experiencing Other” rather than simply “Other” is intended to reflect that there may be other possible forms of “otherness,” such as “logical otherness,” which are not given through empathy. The otherness given through empathy will always be ‘experiencing otherness’, for, as indicated in section 2.1, empathy is simply the comprehension of foreign experience (Stein, [1919/1964] 1989, p. 6).

9 Note that while participants in this study may have shown “compassion,” they still followed through and applied the maximum voltage. This does not, however, indicate that participants did not genuinely sympathize, only that they did not overtly display sympathy by refusing to continue.

10 Stein indicates that while voluntary movement is a key aspect of ascribing Otherness to an entity, it is not strictly necessary; for instance, we can empathize, in a limited sense with plants, and recognize them as “alive,” without ascribing them consciousness or states such as pain and pleasure (Stein, 1964, p. 69).

11 For a discussion of intentions and volitions, see (Adams and Mele, 1992). The focus here will lie on intentions and the will—as Adams and Mele argue, “volition” does not seem to add much to the ‘intention’ concept.

12 See (Seibt, 2017) for a discussion of “as if,” and (Dennett, 1995) for a discussion of humans engaging with objects in this manner.

13 Cf. (Coeckelbergh, 2011; Gunkel, 2012).

14 One might be concerned with a situation in which we experience the robot as Other, but also know that it is *not* an Other (i.e., non-experiencing). Indeed, such a case appears similar to when I have a fear that I know to be irrational, for instance, when I experience fear with respect to the monster in my closet, despite knowing there is no monster in the closet. Such fear is genuine as an experience but ought not dictate our actions, given its irrationality. However, the cases are not actually the same—in the case of fearing the monster while knowing it does not exist or that the fear is unfounded, not letting the fear dictate my actions leans more towards being a virtuous act than a vicious one. In overcoming that fear I practice the process of being courageous, whereas in ‘overcoming’ my perception of the robot as an experiencing Other I am practicing a vicious process, namely dehumanization, as it is discussed in the remainder of this section. Thanks to an anonymous reviewer for raising this concern.

15 Indeed, dehumanization seems to be a failure to recognize the Other which is given to us *as* an Other. In this sense, it is relatable to the preceding discussion of why we may be more inclined to empathize with some objects than others. If our previous classifications of entities as Other/non-Other influences our future classifications as such, then it is sensible that entities such as social robots, which do not fit neatly into our existing categories may be experienced as Other or non-Other with a greater degree of variation than objects that have more stable categorizations as non-Other (e.g., rocks).

16 There are, of course, exceptions (e.g. (Leite et al., 2014)).
